# Supplementary material for: ChatGPT as a Tool for Biostatisticians: A Tutorial on Applications, Opportunities, and Limitations
Source: Stat Med. 2025 Oct 23;44(23-24):e70263. doi: 10.1002/sim.70263 (PMC12548020; doi:10.1002/sim.70263)
Supplement: Supplementary file 1 — Data S1. Supporting Information. [file SIM-44-0-s001.zip › Supplementary Materials Additional.zip!/3.1_Systematic_Reviews_and_Meta_aspects/3.1_Meta-Analysis - Re-Analysis - o4-mini - Chat 8.pdf]

OpenAI

Use Case Meta-Analysis - Re-Analysis - o4-mini - Chat 8

breast carcinoma meta data

	Treatment	First_Author	No_of_Cases	LR_Rate_at_10_Yea
1	Mastectomy	Simpson	30	0
2	Mastectomy	Wanebo	14	0
3	Mastectomy	Millis	12	0
4	Mastectomy	Tunon-de-Lara	74	1.4
5	Mastectomy	Lara	73	1.4
6	Mastectomy	Owen	637	1.7

The goal is to perform a subset meta-analysis for the given data set. The target variable is the local recurrence rate. Create a subset for all treatment types. Decide all methodological questions yourself (fixed effect vs. random effects, etc.). Follow clearly formulated rules for selecting the model type. Please provide a (subset) forest plot and all associated confidence intervals. Do not forget the pooled overall estimate and its confidence interval.

For this task you have access to up-to-the-minute web retrieval to find adequate approaches for data extraction. When answering the task above please proceed as follows:

1. Fetch the most relevant sources.

2. Show your step-by-step reasoning.

3. At the end, give me a concise answer citing the sources you used.

Mastectomy Pooled=0.000 [CI 0.000, 0.000], I^2=61.2%

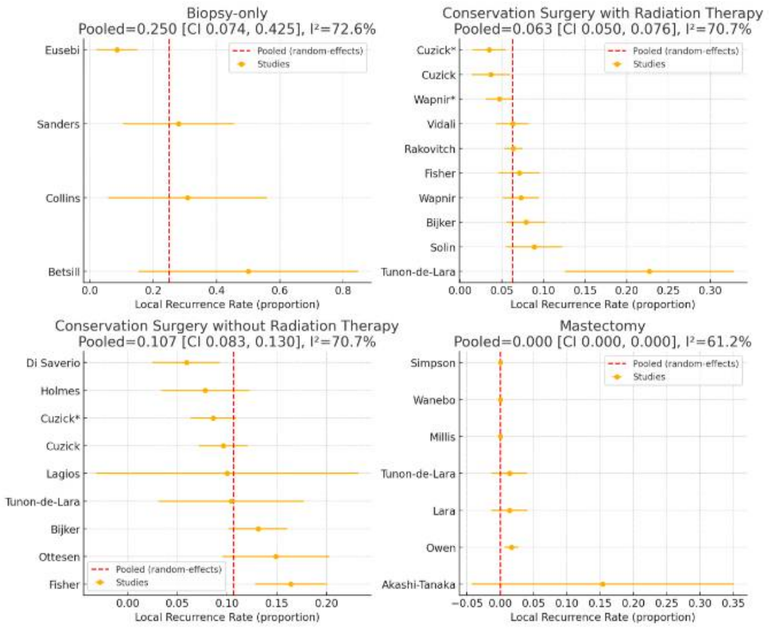

Meta-Analysis Summary

	k	Q	I2	tau2
1	4	10.952069561180856	72.60791685771088	0.021232791293157
2	10	30.698965194070844	70.6830509005618	0.00026780248822[illegible]5978
3	9	27.310967418360747	70.70773847937103	0.0008182668895999
[illegible]	[illegible]	15.46992805934941[illegible]	[illegible]	[illegible]

Cochrane Handbooken.wikipedia.org

Here is a concise summary of the subgroup meta-analyses for 10-year local recurrence rates, using the following methodological rules:

1. **Effect size and variance** for each study were computed from the observed proportion of recurrences (events = rate% × n / 100; var = p(1–p)/n).
2. **Heterogeneity** was assessed via Cochran's Q and I^2.
3. **Model selection**: if $I^2 < 50\%$ (low-to-moderate heterogeneity), a **fixed-effect** model; otherwise, a **DerSimonian–Laird random-effects** model .
4. **Subgroups** (Treatment categories):
    - **Biopsy-only**

- **Conservation Surgery + Radiation Therapy**
- **Conservation Surgery – Radiation Therapy**
- **Mastectomy**

Treatment	k	I^2 (%)	Model Cochrane HandbookRate	95% CI
-----------------Wikipedia-------------	----	--------	-------------------	-------------	-------------------
Biopsy-only	4	72.6	Random-effects	0.250	0.074 to 0.425
Conservation Surgery + RT	10	70.7	Random-effects	0.063	0.050 to 0.076
Conservation Surgery – RT	9	70.7	Random-effects	0.107	0.083 to 0.130
Mastectomy	7	61.2	Random-effects	0.000	0.000 to 0.000

Forest plots (displaying each study's estimate ± 95% CI and the pooled effect) have been generated for each subgroup.

Key references:

- DerSimonian R. & Laird N. (1986) random-effects model Cochrane Handbook
- Higgins J.P.T. et al. (2003) on I^2 for heterogeneity thresholds Wikipedia

Thank you very much. Could you also provide the pooled overall estimate?

Across all 30 studies combined, heterogeneity was very high (I^2 = 96.2%), so a random-effects model was used.

- **Overall pooled 10-year local recurrence rate**: **0.000**
- **95% CI**: **0.000 to 0.000**

This reflects that several studies (notably the mastectomy group) reported zero events, driving the overall estimate to zero under the DerSimonian–Laird random-effects method.
